# Unlocking the power of nanomedicine: the future of nutraceuticals in oncology treatment

**DOI:** 10.3389/fnut.2023.1258516

**Published:** 2023-11-17

**Authors:** Madhav Singla, Smriti, Saurabh Gupta, Prateek Behal, Sachin Kumar Singh, Subham Preetam, Sarvesh Rustagi, Jutishna Bora, Pooja Mittal, Sumira Malik, Petr Slama

**Affiliations:** ^1^Chitkara College of Pharmacy, Chitkara University, Rajpura, Punjab, India; ^2^Department of Pharmacology, Chameli Devi Institute of Pharmacy, Indore, Madhya Pradesh, India; ^3^School of Pharmaceutical Sciences, Lovely Professional University, Phagwara, Punjab, India; ^4^Faculty of Health, Australian Research Centre in Complementary and Integrative Medicine, University of Technology Sydney, Ultimo, NSW, Australia; ^5^Institute of Advanced Materials, IAAM, Ulrika, Sweden; ^6^School of Applied and Life Sciences, Uttaranchal University, Dehradun, Uttarakhand, India; ^7^Amity Institute of Biotechnology, Amity University Jharkhand, Ranchi, Jharkhand, India; ^8^Department of Biotechnology, University Center for Research & Development (UCRD), Chandigarh University, Mohali, Punjab, India; ^9^Laboratory of Animal Immunology and Biotechnology, Department of Animal Morphology, Physiology and Genetics, Faculty of Agri Sciences, Mendel University in Brno, Zemedelska, Brno, Czechia

**Keywords:** Nutraceuticals, cancer, nanotechnology, bioavailability, herbal active compounds

## Abstract

Cancer, an intricate and multifaceted disease, is characterized by the uncontrolled proliferation of cells that can lead to serious health complications and ultimately death. Conventional therapeutic strategies mainly target rapidly dividing cancer cells, but often indiscriminately harm healthy cells in the process. As a result, there is a growing interest in exploring novel therapies that are both effective and less toxic to normal cells. Herbs have long been used as natural remedies for various diseases and conditions. Some herbal compounds exhibit potent anti-cancer properties, making them potential candidates for nutraceutical-based treatments. However, despite their promising efficacy, there are considerable limitations in utilizing herbal preparations due to their poor solubility, low bioavailability, rapid metabolism and excretion, as well as potential interference with other medications. Nanotechnology offers a unique platform to overcome these challenges by encapsulating herbal compounds within nanoparticles. This approach not only increases solubility and stability but also enhances the cellular uptake of nutraceuticals, allowing for controlled and targeted delivery of therapeutic agents directly at tumor sites. By harnessing the power of nanotechnology-enabled therapy, this new frontier in cancer treatment presents an opportunity to minimize toxicity while maximizing efficacy. In conclusion, this manuscript provides compelling evidence for integrating nanotechnology with nutraceuticals derived from herbal sources to optimize cancer therapy outcomes. We explore the roadblocks associated with traditional herbal treatments and demonstrate how nanotechnology can help circumvent these issues, paving the way for safer and more effective cancer interventions in future oncological practice.

## 1 Introduction

Cancer, a broad term encompassing a diverse array of diseases, has the potential to manifest in various anatomical sites within the human body. The onset of cancer is initiated by the conversion of healthy cells into tumor cells through a complex and sequential series of events, typically advancing from a pre-cancerous anomaly to a malignant neoplasm. Cancer, a prevalent ailment, stands as a prominent contributor to global mortality rates, responsible for approximately 10 million fatalities in the year 2020 according to the World Health Organization (WHO) (1). In the United States, recent data from the American Cancer Society reveals that there were 1,918,030 newly diagnosed cases and 609,360 cancer-related deaths in 2022 (2). The acquisition of malignancy by ordinary cells necessitates the prior occurrence of aberrant changes commonly referred to as hyperplasia and dysplasia. Hyperplasia is a pathological condition that is characterized by a significant increase in cell count, while maintaining normal features. In contrast, dysplasia involves cells taking on abnormal phenotypic properties. It is imperative to acknowledge that the occurrence of hyperplasia and dysplasia do not directly result in cancer (3). Generally, early cancer detection and appropriate treatment enhance the likelihood of recovery and survival. The determination of an appropriate treatment modality for cancer is contingent upon several factors, including the specific type and stage of malignancy. A range of therapeutic options including chemotherapy, surgery, radiotherapy, hormonal therapy, targeted therapy, and more. Currently, utilizing a combination of treatments is particularly advantageous for maximizing effectiveness and achieving optimal results (4). Nevertheless, each treatment has its own side effects on patients. Thus, oncologists are faced with the critical task of judiciously selecting the most appropriate treatment option, considering the delicate balance between potential benefits and associated risks (5).

Nutraceuticals, as an emergent field of study, have garnered considerable interest in the past few years owing to its possible implications in cancer therapy. These bio-active compounds, derived from food products and often infused into dietary supplements, have shown promising results in various pre- clinical and lab studies. Current treatments available in the domain of herbal and nutraceuticals range widely from traditional plant-based remedies to vitamins, minerals, and other nutritional supplements. For instance, Echinacea is commonly used to prevent or treat colds, while *Ginkgo biloba* is believed to improve memory. Turmeric, rich in curcumin, is used for its anti-inflammatory properties and many multivitamins are utilized to supplement dietary nutrition (6, 7). However, there are significant limitations to these treatments including unreliability stemming from their bioavailability variations, inherent side effects such as hair loss, nausea, pain, etc. and likely disease recurrence deems them less reliable unless used under vigilant medical supervision (8). Based on research, it has been projected that the nutraceutical business would see substantial expansion, with global valuation expected to exceed $275 billion by the year 2021. The nutraceutical industry surpassed these figures, reaching a value of $382 billion, and further increased to $412 billion in 2020. It is projected that by the year 2024, the market has a high likelihood of reaching a value of around $340 billion. It is estimated that the nutraceuticals business will see a compound annual growth rate of 7.2% over the period from 2016 to 2024 (6, 9). Nanoscale drug delivery systems might offer advantages such as enhanced efficacy, reduced side effects, and improved patient compliance. The foremost advantage nanotechnology offers in drug delivery systems includes the greater bioavailability of therapeutic entities (10). Nanoscale particles have a higher surface area to volume ratio, fostering increased dissolution rate, and therefore, higher bioavailability. Nanocarriers can transport therapeutics across biological barriers that are ordinarily impenetrable for larger particles. Moreover, the enhancement in target specificity is another salient feature offered by nanoscale drug delivery systems. This is accomplished by functionalizing nanoparticle surfaces with molecules that specifically bind to target cells or receptors - an approach commonly referred to as ‘active targeting’. Its ability to manipulate particles at an atomic level ensures targeted therapy, thereby minimizing side effects associated with traditional cancer treatments. The core principle involves utilizing nanoparticles laden with nutraceutical compounds that effectively target tumor cells without affecting healthy ones (11). Additionally, nutraceuticals being naturally obtained entities are considered safe and are highly regarded for their pharmacological attributes. They display lesser adverse effects, which when combined with Nanotechnology can help conquer bioavailability problems often linked to oral administration of drugs (12, 13).

Several mechanisms through which nutraceuticals modulate cellular pathways implicated in cancer are being elucidated by ongoing research. These include their role in apoptosis or cell death, prevention of angiogenesis - the formation of new blood vessels that supply nutrients to tumors – and inhibition of metastasis, a process through which cancer cells migrate and spread across the body (14). In this review, we discuss how nutraceutical phytoconstituents are effective in combating cancer due to their unique properties and ability to target cancerous cells. In the subsequent sections, we explored the background and significance of nutraceuticals in healthcare, followed by an in-depth analysis of selected phytoconstituents with anti-cancer properties, and concluding with potential future innovations in this field.

## 2 Approaches for the research methodology

All of the information included in this article was obtained from multiple sources, including Science Direct (Elsevier), PubMed, Medical Subject Headings (MeSH), and Google scholar by using the combination of keywords: “Cancer,” “nutraceutical,” “phytoconstituents,” “nanoformulation,” “biomarkers” and “*in vitro/vivo studies*.” The article titles and abstracts were examined to determine the search phrases used to select the papers. A portion of the articles were manually screened, and those that were irrelevant or duplicates were disregarded. Only papers that satisfied the inclusion criteria were used in the research. Inclusion criteria involved were: (a) an original publication from a peer-reviewed journal; (b) management of several biomarkers (c) evaluation of *in-vitro* biological studies and/ or *in vivo* pharmacological activities of various phytoconstituents and even their nanoformulations. Exclusion criteria were (a) studies that were not pharmaceutical or pharmacological applications and (b) articles authored in any language other than English. Following filtering and screening, 245 published articles that met the review’s inclusion criteria were included. We also evaluated the cited publications’ references and incorporated them for discussion in the review.

## 3 Basic pathology of cancer

Cancer is a complex disease characterized by uncontrolled cell growth owing to alterations in gene expression within the genome. This disruption in the delicate equilibrium between cell proliferation and results in an augmentation of the cellular population capable of infiltrating adjacent tissues and disseminating to remote anatomical sites inside the organism, causing significant illness and, if left untreated, mortality for the individual affected (15). At the molecular level, various types of cancers share certain characteristics. This indicates that the underlying biochemical changes responsible for the development and advancement of malignancy may arise from a similar, albeit not identical, set of modifications in gene expression. The fundamental challenge in understanding the pathology of cancer lies in the recognition and comprehension of intricate biological regulatory mechanisms as well as the deciphering of metabolic activities occurring at the molecular, cellular, organ, and systemic levels. Hence, the pathology of cancer can be determined by examining the following areas (16).

### 3.1 Genetic mutation

Genetic damage is a fundamental aspect of tumor development, wherein somatic mutations in genes have been detected in approximately 90% of cases. In addition, germline mutations in genes derived from the ectoderm, mesoderm, and endoderm have been identified in approximately 20% of human cancers, and a small proportion of neoplasms exhibit both somatic and germline mutations (17, 18). These observations suggest that excessive expression of certain genes may possess the capacity to initiate the development of cancer. This can occur through various mechanisms such as gene amplification, deletion or insertion, promotion of gene translocation, and regulatory miRNA modulation (19). MicroRNAs (miRNAs) have emerged as a noteworthy mechanism involved in the control of gene expression. In the realm of cellular biology, it is remarkable to note that over a thousand distinct types of miRNAs are expressed within the vast majority of cells. Principally, they exhibit an affinity for messenger RNA molecules and facilitate their degradation, consequently impeding the process of translation and impacting the regulation of gene expression. Certain miRNAs have been linked to various types of human neoplasms, specifically colorectal cancer and chronic lymphocytic leukemia. However, further research is required to fully understand the significance of miRNAs in neoplasia pertaining to veterinary medicine (20). Alterations in the genetic makeup of proto-oncogenes can cause disruptions to normal cellular functions, leading to the initiation of tumor growth. Following this alteration, the proto-oncogene undergoes a transformation and is referred to as an oncogene. Over 100 oncogenes have been recognized thus far, and their prevalence continues to expand as genetic investigations of neoplasms continue (21). Considering the fact that numerous proteins encoded by proto-oncogenes play crucial roles in promoting cellular proliferation (22). Phenotypic manifestation of the initial indicators of cancer, known as Sustaining Proliferative Signaling (oncogenes and proto-oncogene), is commonly identified as an elevation in the rate of cell division, specifically mitotic activity. Various techniques exist for evaluating cell proliferation, such as the use of proliferating cell nuclear antigen (PCNA) or Ki67 staining, which may offer a more easily interpretable alternative to mitotic counting (20).

Mutations observed in the cancer genome were also acquired through prolonged exposure to both endogenous and exogenous mutagens. The presence of carcinogens, such as those found in tobacco smoke, aflatoxins, and radiation, has been linked with the growth of lung, liver, and skin cancers, respectively. Several genes that have been extensively studied and have been identified as bearing mutations in cancer include TP53, RB1, EGFR, and KRAS (19). These genes are commonly mutated in different types of cancer, but they are relatively rare and may be specific to only one type of cancer. Chromosomal transformations and gene fusions are frequently observed genomic errors in cancer. In the context of prostate cancer, it is worth mentioning that there are frequently observed gene fusions. One such example involves the ERG gene, which leads to the formation of a TMPRSS2-ERG fusion gene. Additionally, ETV1 has been implicated, resulting in the formation of the TMPRSS2-ETV1 fusion gene (23, 24).

### 3.2 Absence of tumor suppressing genes and apoptosis in cancer cell

Healthy cells constantly analyze their internal and exterior environments before cell division to make sure circumstances are favorable for mitosis. Whether or not a cell proliferates, quiescence, reaches a post-mitotic stage, or experiences self-destruction is determined by signals from that environment. Genes known as tumor suppressors are essential for regulating healthy cell proliferation. They act as the barriers that stop cell division. The biological control over cell growth is lost when these genes are inactivated. To keep cell proliferation under control, only one tumor suppressor gene is sufficient (15, 25).

**Figure 1:**
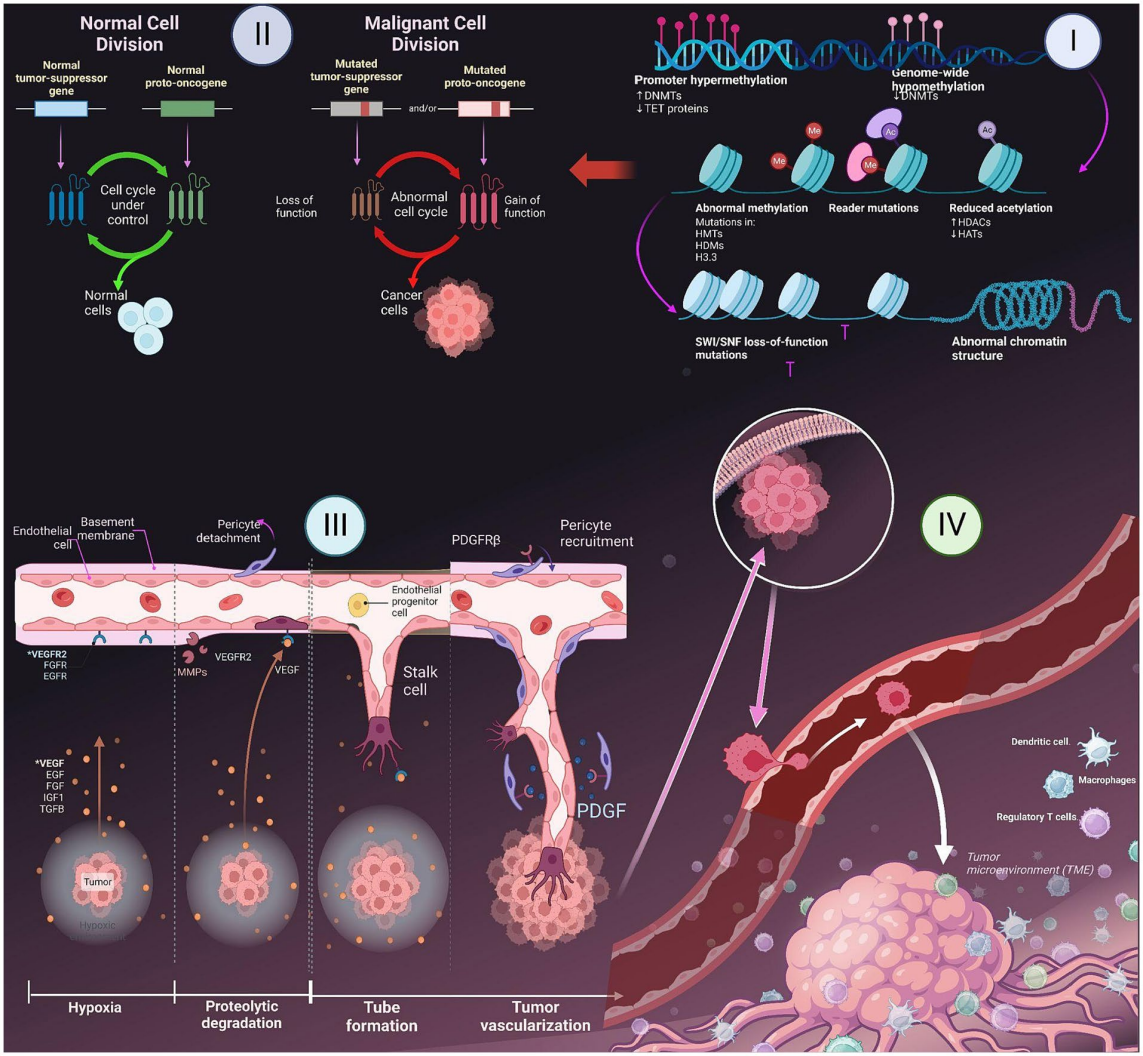
Major hallmarks of cancer- I. Epigenetic changes in gene; II. Role of tumor suppressing genes and apoptosis; III. Induction of angiogenesis; IV. Metastasis to other parts of the body.

The cell cycle encompasses a series of distinct and biochemically intricate phases, each serving to prepare the cell for the process of division. In healthy cells, the initial phase of the cell cycle is referred to as G phase. During this phase, which spans approximately 3 to 8 h, the cell actively engages in the formation of RNA. Subsequently, in the S phase, which typically lasts from 6 to 12 h, the cell dedicates its resources to the replication and development of DNA. Afterwards, the G2 phase ensues, wherein the diploid chromosomes undergo their finalization, culminating in the M phase, a pivotal stage wherein mitosis occurs within a span of 1 hour (26). The DNA that undergoes radiation-induced damage within a compromised cell has the potential to repair before the transfer of any changes to the genomes of its daughter cells during their transition between different phases. In tumor suppressor gene-deficient cells, the presence of genomic damage is frequently observed without repair mechanisms as shown in Figure 1 (27). This often leads to a state of genomic instability within the cells, which in turn increases chances of occurrence of oncogenic events. When a cell encounters stress, such as DNA damage or hypoxia, the p53 protein undergoes a process of assembling into a stable tetrameric complex. This complex then endeavors to manage the cell cycle by activating specific genes that are responsible for halting cell cycle, facilitating repairing of DNA, or triggering apoptosis (15).

Tissue homeostasis is a delicate equilibrium between cellular proliferation and apoptosis, wherein the cellular population within that specific tissue remains relatively stable. Cancer cells have developed various mechanisms to evade the process of apoptosis. The prevailing approach frequently entails alterations in the p53 tumor suppressor gene, leading to the depletion of pro-apoptotic regulators. Inhibition of the p53 protein is observed in over 50% of human cancers, cells that have had genetic damage have impaired escape pathways that would typically induce programmed cell death in healthy cells. The tumors demonstrate an increased expression of the BCL2 gene, leading to the suppression of programmed cell death, also known as apoptosis. The capacity of oncoproteins, like BCL2, to impede the pathways leading to cell death, may grant cells (with genetic damage) the ability to evade the mechanisms that typically trigger apoptosis in healthy cells (15, 28).

### 3.3 Angiogenesis

Angiogenesis, the formation of new blood vessels, is a process that is regulated by a delicate balance of pro-angiogenic and anti-angiogenic signaling in both normal tissue and neoplastic masses (25). Tumor cells have the ability to release growth factors, including VEGF-A and different types of FGF. These growth factors can either accelerate the vessels while assisting stroma within tumors and induce other cells to secrete angiogenic factors, which further promote the growth of blood vessels in the tumor microenvironment (29). These factors have the ability to disperse throughout the surrounding tissues and bind to receptors located on the endothelial cells of preexisting blood vessels. This interaction ultimately triggers the growth of these blood vessels. The dynamic interplay between endothelial cells and tumor cells results in the release and subsequent stimulation of a diverse array of proteolytic enzymes, including MMPs as shown in Figure 1. These enzymes play a crucial role in breaking down the basement membrane and extracellular matrix. Edema formation occurs as a result of increased interstitial pressure, which is primarily attributed to the presence of permeable vessels and the absence of lymphatics to effectively remove the leaked fluid. The presence of edema and subsequent increase in pressure can lead to the compression of blood vessels within the tumor, resulting in local blood flow obstruction. Due to the presence of irregular blood flow and perfusion, there are localized regions within the body that experience a reduced supply of oxygen (hypoxia) as shown in Figure 1 or a complete lack of oxygen (anoxia). The degradation of the basement membrane facilitates the movement of activated endothelial cells, which are prompted to multiply due to the presence of growth factors, towards the tumor. Integrin molecules, such as avẞ3-integrin, play a crucial role in facilitating the forward movement of sprouting new blood vessels. The endothelial cells facilitate the deposition of a novel basement membrane and actively secrete growth factors, including platelet-derived growth factor (PDGF) (30). These growth factors play a crucial role in attracting supporting cells to promote the stabilization of the nascent vessel.

### 3.4 Metastasis

Metastasis is a biological phenomenon characterized by the detachment of a cancer cell from the initial tumor, migration to an alternate site through the bloodstream, and establishment of a secondary tumor (31). Due to its complexity and limited efficiency, only a few cells have the ability to execute the metastatic process. Throughout the progression of tumor development, it is possible for a metastatic subclone to emerge inside a malignant tumor. The disruption of intercellular adhesion is caused by the compromised functionality of cell adhesion factors like E-cadherin. There is a two-phase process that facilitates the migration of metastatic cells into the basement membrane (32). Initially, metastatic cells adhere to the basement membrane by means of specific receptors such as laminin and fibronectin. Subsequently, these cells release hydrolytic enzymes, namely proteases, which aid in the destruction of the basement membrane. The subsequent phase involves locomotion, in which the tumor cells have the ability to move into the extracellular matrix with the assistance of substances that are released by both the tumor cells themselves and inflammatory cells present in the host, which are macrophages. Once cancer cells have metastasized, they penetrate either a lymphatic or a blood vessel. The concept of intravasation involves the entry of tumor cells into the circulatory or lymphatic systems. This procedure necessitates the initial adhesion of cancer cells to vessel’s basement membrane and then its subsequent degradation. The process of extravasation is assisted by the enhanced permeability of newly created, deformed blood vessels inside tumors. After tumor cells permeate the bloodstream, they come into contact with several host cells that participate in the immune-mediated eradication of tumor cells. In order to ensure their survival, tumor cells must successfully escape the vigilant surveillance exerted by the immunological response of the host organism (32, 33).

## 4 Various nutraceutical phytoconstituents being employed for anticancer therapy

Nutraceuticals and phytoconstituents found in certain foods have been shown to have potential health benefits beyond simple nutrition. They have gained popularity for their potential role in preventing and treating cancer. They are known to have antioxidant and anti-inflammatory properties, which can help combat the oxidative stress and inflammation that contribute to cancer development. In particular, phytoconstituents, or the biologically active compounds found in plants, have shown promise in mitigating cancer growth and progression. The use of nutraceuticals as complementary medicine has the potential to enhance the effectiveness of conventional treatments while minimizing treatment-related side effects. Some of the most promising nutraceuticals and phytoconstituents in anti-cancer research include curcumin, resveratrol, green tea polyphenols, and sulforaphane (34, 35). In recent years, there has been an increasing interest in studying the potential of nutraceuticals as anti-cancer agents. While research is still ongoing, early studies have shown promising results, moreover nutraceuticals an exciting and exploring area of research for cancer treatment and prevention. In that era some of the promising plant based phytoconstituents obtained from nutraceuticals.

### 4.1 Resveratrol

Peanuts, grapes, pistachios, red wine, blueberries, and cranberries are common sources of the natural polyphenol resveratrol (trans-3,5,4′-trihydroxystilbene). It possesses a molecular formula of C14H12O3 and a molecular weight of 228.25 g/mol. The ethylene bridge connects two aromatic rings in this molecule. Both C3 and C5 in ring A are hydroxyl groups, whereas C4′ in ring B is the only -OH group. It photo-isomerizes its naturally occurring trans form (E configuration) to its cis form when exposed to UV or visible light (36). Since they share their structural similarity to estrogen, this phytoestrogen can interact with estrogen receptors, which activates their downstream signaling pathways, altering kinase activity, transcription of mRNA, and other critical cellular processes. In a variety of malignancies, it inhibits tumor growth and kills cancer stem cells (37). Resveratrol’s ability for delaying/blocking the beginning, development, and advancement of tumor, make it a significant factor in cancer therapy. It is known to exert a noteworthy influence on the prevention and advancement of cancer by regulating diverse cellular signaling pathways, as outlined in Table 1. This phytoconstituent functions as a robust antioxidant via the Nrf2/HO-1 pathway and as an anti-inflammatory agent by suppressing the expression level of TLR-4, NF-κBp65, and the release of proinflammatory cytokine. These actions impede the advancement of tumors, diminish the likelihood of cancer, and promote metastatic dissemination (83–85). In addition, it has been observed to activate genes that suppress tumor growth, including PTEN, caspase-3, and p53 (86). Furthermore, it has been found to induce apoptosis in cancer cells by inhibiting P13K/Akt and Bcl-2, while elevating the Bax (87). These molecular mechanisms are known to be involved in tumor invasion, development, and angiogenesis. The findings of various studies indicate that it has potential to cause halt cell cycle in the G0/G1 phase. This effect is attributed to the downregulation of cyclin D1, cyclin-dependent kinase (CDK)4 and CDK6 levels and the upregulation of p21 and p27 and CDK inhibitors expression levels (88).

**Table 1:** Current anti-cancerogenic research studies of various phytoconstituents.

S.No.	Phytoconstituent	Type of cancer	Model	Dose	Outcome	Reference
1.	Resveratrol	Oral	*in-vitro* study on HOSCC	2–100 μM	It lowers the level of E-FABP and SREBP1, which inhibits cell multiplication and leads to autophagic cell death	Fukuda et al. (38)
		Breast	*in vivo* study on female Sprague Dawley rats	20 mg/kg	Transdermal patches of resveratrol lowers the volume of tumor and serum CA 15–3	Gadag et al. (39)
		Colon	*in-vitro* study on Caco-2 cell line	–	It lowers the level of γ-H2AX and induces apoptosis	Wang et al. (40)
		Lung	*in-vitro* study on LCSC	0, 12.5, 25, 50 μM	It impaired the stimulation of Wnt/β-catenin and reduces lung cancer	Xie et al. (41)
		Liver	*in-vitro* experiment on MHCC97-H cell line	0–200 μM	It lowers cyclin D, MAPK, Akt and p38, decreasing hepatic carcinoma	(42)
		Prostate	*in-vitro* experiment on PC- 3 cell line	100 μM	It suppresses mTOR and induces autophagy which blocks migration of cells	Vidoni et al. (43)
2.	Curcumin	Oral	*in-vitro* study on TSCCF cell lines	1,000, 500, 250, 125, 62.5, 31.25, 15.625, 0 μg mL^−1^	CUR demonstrates a higher degree of specificity towards oral cancer cells and induces cell death causing reduction in cell viability	Hussein et al. (44)
		Breast	*in-vitro* study on MCF-7 breast cancer cells	5-25 μM (different concentration of Fe3O4@SiO2- curcumin, Fe3O4@SiO2-piperine and Fe3O4@SiO2-curcumin/piperine)	Magnetic nanoparticles possess anti cancer properties against breast cancer	Rezaeidian et al. (45)
		Colon	Molecular docking study via AutoDock Tools 1.5.7	–	The findings from the molecular docking analysis indicate that curcumin exhibited an innate binding affinity towards key target proteins such as CDK2, HSP90AA1, AURKB, CCNA2, TYMS, CHEK1, AURKA, DNMT1, TOP2A, and TK1. It disrupts the processes of cellular proliferation and programmed cell death in malignant cells	He et al. (46)
		Lung	*in-vivo* study on Male BALB/c nude mice	5 mg/kg	The inhibition of TrxR leads to the induction of apoptosis and ferroptosis that is reliant on on ROS	Liu et al. (47)
3.	Quercetin	Oral	*in-vitro* experiment on CAL27 cell line	20, 40, 60 or 80 μM	It inhibits the uptake of glucose and its breakdown, along with the expansion of cancer cells	Hu et al. (48)
		Breast	*in-vitro* study on MCF-7 cell line	4 mg/kg	Their nanoformulation lowers the multiplication of cancer cells by abolishing PCNA gene	Elsayed et al. (49)
		Colon	*in-vitro* study on HT-29 cell line	40–180 μM	Its mechanism of action involves upregulating the p53 gene and downregulating cell viability via intensifying apoptotic activity resulting from the upregulation of ESR2 gene	García-Gutiérrez et al. (50)
		Liver	*in-vitro* study on HepG2 cell line	0, 5, 10, 20, 30, 40 μM	Quercetin promotes upregulation of tumor-suppressing genes which initiates apoptotic cell death by disruption of the YY1-p53 interaction	Guan et al. (51)
		Ovarian	*in-vitro* study on PA-1 cell line	0–200 μM (75 μM is effective concentration)	It leads to upregulation of cyt c, caspase-9 and 3 which initiates cell death and a reduction in ROS levels	Teekaraman et al. (52)
4.	Beta lapachone	Breast	*in-vitro* study on MDA-MB-231 cell line	0, 2, 3 and 4 μM	It triggers cell death in cancer cells by stimulating PKA through ROS formation	Zada et al. (53)
		Liver	*in-vitro* experiment on Human HCC cell lines	0–10 μM	It triggers apoptosis in via NQO1 which significantly decreases ROS and enhanced activity of PARP1	Zhao et al. (54)
		Gastric	*in-vitro* experiment on AGS cell line	0–4 μM	Cell death is induced by the reduction of Bcl-2/Bax and PI3K/Akt signaling pathways.	Yu et al. (55)
		Pancreatic	-	-	It induces cell death by elevating the level of NQO1	Qadir et al. (56)
	Epigallocatechin gallate	Oral	*in-vitro* study on SAS cells	100 mM	The study observed an increase in the release of histamine and a reduction in the amount of H1R	Kon et al. (57)
		Breast	*in-vitro* study on MCF-7 cell	0.5–20 μg/mL	The induction of cell death and disruption of cell cycle development at the G2/M phase was observed. It was also observed that it suppressed the amount of miR-25 and upregulated the amount of PARP, pro-caspase-3 and 9 proteins	Zan et al. (58)
		Sarcoma	*in-vitro* study on RMS cell line		The intervention resulted in a decrease in cellular multiplication and a downregulation of the Hedgehog pathway	Mayer et al. (59)
		Colorectal	*in-vitro* studies on HCT116 and HT‐29 cell line	0–1 μM	It suppresses the PI3K/Akt/mTOR pathway and triggers cell death in cancer cells	Khiewkamrop et al. (60)
		Liver	*in-vivo* study on Wistar rats (male)	300 mg/kg	The observed effect involves elevated antioxidant activity, leading to a concomitant decrease in the amount of MDA and a rise in GSH levels. In addition, there was a notable decrease in the manifestation of TNFα, NFjβ, IL1β, and TGFβ	Mostafa-Hedeab et al. (61)
6.	Ellagic acid	Breast	*in-vitro* study on MCF-7 cell line	0–250 μM	It effectively inhibits CDK6, thereby impeding both the advancement of cell cycles and expansion	Yousuf et al. (62)
		Lung	*in-vitro* studies on HOP62 and H1975 cells	10–100 μM	Autophagy induction was seen as a result of downregulation of p-Akt, p-mTOR, p-P70S6K, and CIP2A.	Duan et al. (63)
		Liver	*in-vivo* study on Female wild-type FVB/N mice	150 mg kg − 1 and 300 mg kg − 1	The mechanism of action involves deregulation of the AKT/mTORC1 and impairment of SREBP-1/FASN axis	Zhang et al. (64)
		Prostate	*in-vitro* study on PCa cell line	5, 10, 20, 40, 80 and 160 μM	The observed effect was a decrease in the amount of MDM2 and a rise in p53 protein synthesis	Mohammed Saleem et al. (65)
		Gastric	*in-vitro* study on AGS cell line	100–3.125 μg/mL	The observed effect was a drop in MMP-2 and 9 levels, as well as a restriction on cell movement and initiation of cell death	Cheshomi et al. (66)
7.	Daidzein	Breast	*in-vitro* experiments on MCF-7 and MDA-MB-231 cell lines	25–100 μM	The results indicated that it elicited a significant reduction in cellular proliferation and viability	Medeiros et al. (67)
		Prostate	*in-vitro* experiement on LNCaP cell line	-	The study revealed a decline in testosterone levels and a boost in p53	Sivoňová et al. (68)
		Lung	*in-vitro* study on H1299 cells	10 μM	Through the production of caspase 9 and the enhancement of tumor suppressing genes, it triggers apoptosis	Liu et al. (69)
		Ovarian	*in-vitro* study on SKOV-3 cell	0, 0.78, 1.56, 3.12, 6.25, 12.5, 25, 25, 50, 100 and 200 *μ*M	A significant decrease in MMP was detected, along with an elevation in the levels of cytochrome c, Bax, and cleaved forms of caspase-3 and − 9.	Hua et al. (70)
8.	Dihydroartemisinin	Lung	*in-vitro* studies on XWLC-05 and NCI- H23 cell line	30 mg/kg	The PRIM2/SLC7A11 axis was found to be impeded, resulting in the suppression of multiplication and aggregation, as well as the activation of ferroptosis of cancer cells	Yuan et al. (71)
		Liver	*in-vitro* study on PLC cell lines	2, 5, 7.5, 10, 20, 30, 40 or 50 μM	It suggests that the process efficiently triggers ferroptosis in cancer cells and initiates the unfolded protein responses	Wang et al. (72)
		Prostate	*in-vitro* study on PCa cell lines	0.0048–5 μM	The repression of Axl levels leads to reduced development, movement, and spread, as well as the occurrence of cell death in cancer cells. Additionally, tumor growth is inhibited	Paccez et al. (73)
		Bladder	*in-vitro* study on T24 cell lines	25–400 μM	The anti-cancer activity is mediated through the modulation of the KDM3A and p21 pathway	Wang et al. (74)
9.	Triptolide	Oral	*in-vitro* experiment on OSCC cell line	0–80 mM	It decreases PD-L1 expression in cell line	Kuo et al. (75)
			*in-vivo* experiment on PDTX models (mice)	0.15 mg/kg	It significantly shows anti-cancer properties via regulating PD-L1 expression	
		Breast	*in-vitro* study on MCF-7 and MDA-MB-231 cell lines	0.01, 0.1, and 1 nM	It exhibits a major inhibitory impact on cancer development both *in vitro* and *in vivo*. It down- regulates HMGB1 expression, thereby inhibiting cell proliferation	Jiang et al. (76)
			*in-vivo* experiment on Female BALB/c athymic mice			
		Esophageal	*in-vitro* experiment on KYSE180, KYSE150 cells and *in- vivo* experiment on nude mice	0, 4, 8 nM and 0.15 mg/kg	It inhibits cell multiplication and spread of carcinoma cell lines via alterations in phases of cell cycle causing cell death, both *in vitro* and *in vivo*	Yanchun et al. (77)
		Colon	*in-vitro* study on HT‐29 colon carcinoma cells	0, 25, 50, 75, and 100 nM	Triptolide interferes in development of cancer cells and cell cycle’s progresses. It also exhibits inhibitory effects on establishment of spheroids and movement	Acikgoz et al. (78)
		Lung	*in-vitro* experiment on NSCLC cells and *in- vivo* experiment in male nude mice	0, 12.5, 25 or 50 nM and 400 μg/kg	In both studies, it effectively halt the cell multiplication by downregulating HAS2, HAS3, HA, CD44, and RHAMM via intranasal route in mice	Song et al. (79)
		Prostate	*in-vitro* studies on PCa, LNCaP cells and *in- vivo* study on male nude mice	0.1 μM and 0.4 mg/kg	It possesses inhibitory impacts on cellular multiplication and provokes apoptosis in LNCaP and PCa cell lines whereas reverse the xenografted PC-3 tumor growth *in vivo* as a result of the intervention.	Huang et al. (80)
10.	Genistein	Lung	*in-vitro* studies on NSCLC (H292 and A549) line	0, 10, 20, 40, 80 and 160 μM	It inhibits NSCLC cell viability in a concentration-time-reliant way. It is capable of suppressing these processes such as multiplication, movement, spread, and cell death through its regulatory effect on FOXM1.	Yu et al. (81)
		Liver	*in-vitro* study on HepG2 carcinoma cell line	6.2–100 μM	It has the ability to trigger mitochondrial death and interrupt the cell cycle by deforming ROS.	Zhang et al. (82)

### 4.2 Curcumin

Curcumin is a polyphenol that is a member of the diarylheptanoid class. A yellow pigment made from the rhizomes of the *Zingiberaceae* family plant, *Curcuma longa* (turmeric). With a molecular weight of 368.38 g/mol and the formula C21H20O6, it is a symmetrical molecule. Three chemical elements make up curcumin, according to chemistry: a 7-carbon linker that joins two ortho-methoxy-phenol aromatic rings together is made up of an α,β-unsaturated β-diketone linker. CCM may swiftly function as an electron donor and maintain the structure through the resonance of the tiny electron cloud as a result of its double bond in the chemical framework. It may also participate actively in a broad range of electron-transfer activities (89). The literature proposes that the inclusion of a hydroxyphenyl moiety in curcumin-like compounds, particularly at the 2-position, enhances their chemoprotective properties by facilitating the induction of phase II elimination (90). Its anticancer properties are demonstrated through its ability to hinder cellular transformation, avoid the expansion of tumor cells, and minimize tumor-promoting properties. Additionally, they have demonstrated the ability to elicit apoptotic responses in drug-resistant cells, thereby enhancing the cytotoxic impact of various chemotherapeutic enzymes. The aforementioned entities serve as activators of STAT3, protein kinase B (AKT), AP1, p13/Akt/p53, cytochrome C levels, and lower NF-kB levels (91).

### 4.3 Quercetin

**Figure 2:**
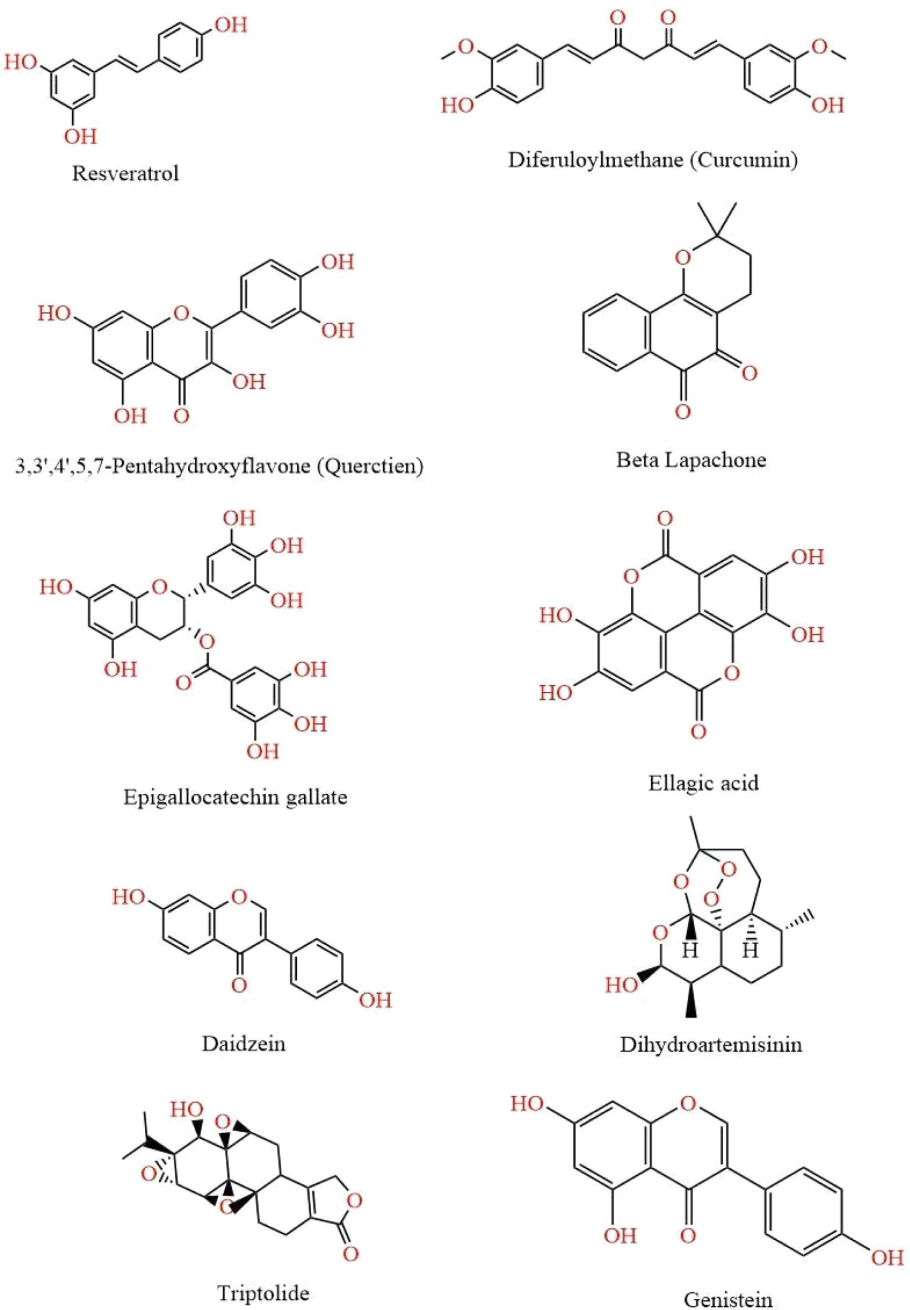
This figure illustrates various chemical structures that have been reported to possess anti-cancer properties. Each compound represents a distinct class of molecules known for their potential in inhibiting cancer cell growth and promoting apoptosis. The structures are Resveratrol, Curcumin, Quercetin, Beta Lapachone, Epigallocatechin Gallate, Ellagic Acid, Daidzein, Dihydroartemisinin, Genistein, and Ttriptolide.

Quercetin, having a chemical formula of C15H10O7 and a molar mass of 302.236 g/mol, is a flavonol derived from polyphenols. They are groups of compounds that include flavonoids and tannic acid and contain multiple phenol units. This compound is primarily obtained from various plant sources such as apples, grapes, capers, berries, and onions (92). It is characterized by presence of a catechol moiety in the B ring and hydroxyl groups at positions 3′ and 5′ in the A ring. It also possesses a 2,3-double bond that is in conjugation with a 4-oxo function located in the C ring. From a structural perspective, the three aforementioned rings are crucial elements of the antioxidant capacity (93). It has demonstrated significant potential in the field of oncology owing to its chemo preventive outcomes as observed in both *in vitro* and *in vivo* models. It exhibits two-phased effects that depend on the dosage administered. At lower doses, it functions as an antioxidant, thereby inducing anti-cancer results at higher doses (94). It has been observed to modulate the NF-κB pathway, which is accountable for the regulating the expression of various genes including interleukins associated with processes such as cancer development, inflammation, and cytoprotection. It possesses the capacity to impede cell replication, trigger cell death, and lead to cell cycle termination via controlling the activity of signaling pathways such as cyclins, pro-apoptotic, PI3K/Akt, and MAPK (95). Furthermore, it was discovered to hinder the phosphorylation of mTOR and Aktser473 while facilitating GSK-3 and STAT stimulation. It also reduced prosurvival cellular proteins including cyclin D1 and c-Myc and cyclin D1 and c-Myc, and initiated the destruction of β-catenin (96) (Figure 2).

### 4.4 Beta lapachone

β-Lapachone is a naturally occurring ortho-naphthoquinone compound with a molecular formula of C15H14O3 and a molecular weight of 242.27 g/mol. It has been extracted from the lapacho tree, which is indigenous to various regions of South America (97). Its molecular structure comprises a benzene ring that confers its anti-tumor, anti-malarial, and antibacterial properties. Additionally, an ortho-quinone ring and a dihydropyran ring are responsible for its anti-Trypanosoma cruzi and anti-TB activity, respectively (98). The antitumor efficacy of β-lapachone has been demonstrated to be majorly associated with stimulation of NAD(P)H: quinone oxidoreductase-1 (NQO1), resulting in the prompt generation of ROS and various modulations of the pathways related to cancer progression and proliferation. Previous research has proposed that it possesses antitumor properties by obstructing topoisomerase I, topoisomerase II, IDO1, telomerase, and HSP 90 (99).

### 4.5 Epigallocatechin gallate

Green tea contains a potent polyphenol called epigallocatechin-3-gallate that is obtained from the leaves of the evergreen shrub *Camellia sinensis*, a member of the Theaceae family (100). The molecular formula of the compound is C22H18O11 and its molar mass is 458.372 g/mol. It features a pyran ring (C) connecting its three aromatic rings (A, B, and D). Heat-shock protein 90 is inhibited by the A ring. Its antioxidant effect evolved from a hydrogen atom transfer involving the hydroxyl groups of the B and D rings (101). These rings are also linked to an *in vitro* suppression of proteasome activity (102). It supports potent anticancer effects via numerous mechanisms. Its anti-cancer effect is attributed to a regulation of ROS generation and inhibition of NF-kB, which is associated with suppression of angiogenesis, migration, and cell viability. It stimulates MAPK activity which enhances its anti-carcinogenic property towards invasion, movement, and cell death of cancer cells. Additionally, it promotes epigenetic modification by suppressing DNA methyl-transferase activity and controlling histone acetylation, which increases cell death (103, 104).

### 4.6 Ellagic acid

Ellagic acid is a phenolic compound which is obtained from seeds, peels and arils of Pomegranate. It is also found in fruits such as grape, pomegranate, strawberry, persimmon, peach, plum, raspberry, nuts including almonds and walnuts, vegetables and wine (105). It is a hydroxybenzoic derivative made from phenolic acids, hydroxyl group and benzene ring, considered the most effective components for lowering free radicals. It is composed of a central structure consisting of fused aromatic rings, to which are attached two acyloxy groups and four free hydroxyl groups. The hydrophilic domain is represented by two lactones and four phenolic groups, whereas the lipophilic domain is represented by four rings. It has a molecular weight per mol of 302.197 and M.P. of 350 degrees celcius (106). It prevents malignancies from binding to DNA and triggering cell death and reduces the formation of tumor cells. It also impairs pathways including inflammation, angiogenesis, and drug resistance that are necessary for tumor development and spread (17). According to research it can be employed with chemotherapy treatments to impede the growth and spread of cancer as well as cure it (107).

### 4.7 Daidzein

Daidzein is commonly present in soybeans and certain other legumes. It is classified as an isoflavone phytoestrogen belonging to the non-steroidal estrogens, which is isolated from Pueraria Mirifica. The molecular formula of daidzein is C21H20O9, with a corresponding molecular weight of 416.4. It is a physiologically active secondary metabolite which belongs to the flavonoid group. Its chemical structure is characterized by 7-hydroxy-3-(4 hydroxyphenyl)- 4H-chromen-4-one (108). It has the ability to trigger cell death and cease the cell cycle in cancerous cells, as well as potentially regulate the synthesis of long noncoding RNA in specific forms of cancer. The phenomenon has been linked to a reduction in the amounts of HER2/neu and PCNA within cancerous growths, which corresponds closely with a more aggressive cancerous cell. Additionally, it has been observed that the levels of ERβ are enhanced, which may confer an inhibitory effect against cancer (109).

### 4.8 Dihydroartemisinin

Artemisinin, an extract of *Artemisia annua* L. Dihydroartemisinin, represents the initial derivative of this compound. It is a chemical compound characterized by a molecular formula of C15H24O5 and a corresponding molecular weight of 284.35 (110). It exerts anti cancer activity through diverse processes, including the inhibition of expansion and angiogenesis. These effects are achieved by decreasing the expressions of PCNA, Cyclin E, Cyclin D1, Bcl-2, and the P13K/Akt pathway. The stimulation of JAK, STAT, and JNK pathways has been observed to induce cell death, enhance immune function, promote autophagy, and trigger ER stress (111).

### 4.9 Triptolide

A functional diterpene triepoxide known as triptolide was discovered having a molecular formula of C20H24O6 and weight of 360.4, through the Chinese herbal plant Tripterygium wilfordii Hook F. It is present in various species of Tripterygium, including *T. hypoglaucum* Hukeda, T. forretii Dials, and T. regelli Sprague et Taketa (112). The butenolide group and the epoxide group are equally essential for its inhibitory property on multiplication of cancer cells, and the C-14 hydroxyl group is an adequate functional section for chemical changes in order to increase its dissolution as well as its biological effects. Its structural alteration is centered on various locations such as the β-hydroxyl group at C14, epoxides, the butanolide, C-5 and C-6, C- 13 isopropyl group, lactone ring and other places. The ability to inhibit multiplication of cancer cells is dependent on the two components: the butenolide group and the epoxide group. Additionally, the C-14’s β-OH group is a viable functional part for chemical modifications aimed at enhancing its dissolution and biological activities (113, 114). The transcriptional inhibition exerted by triptolide is attributed to the suppression of numerous proteins that are crucial in various biological processes such as apoptosis (Bcl-2 and Bcl-xL), cell cycle regulation (cyclins A1, B1, and D1), oncogenesis (HSP70, p21, p27, and RNA polymerase), inflammation, and immunoregulation (interleukins, TNF-a, CD40, and 80) (115).

### 4.10 Genistein

A naturally existing flavonoid called genistein, which is an element of Leguminosae plants, is a phytoestrogen. Therefore, soy products such as soybean (*Glycine max* (L.) Merr.) and Trifolium species are the main sources of it (116). With a molecular weight of 270.239, pure genistein (C15H10O5) is a white powder that is insoluble in water. With the identical phenolic ring foundation and gap between the 4′ and 7’ OH groups as 17-estradiol, it is known as 4′, 5, 7- trihydroxyisoflavone (117). Genistein has been found to exhibit suppressive effects on various cellular components such as interleukins, COX-2, phosphorylated-JNK, ERK1/2, PI3K/Akt and MMP-2, Cdc25A and C and Cdc2. These effects have been observed to lead to the stimulation of cell death and inhibition of cell cycle, angiogenesis and metastasis (118).

**Figure 3:**
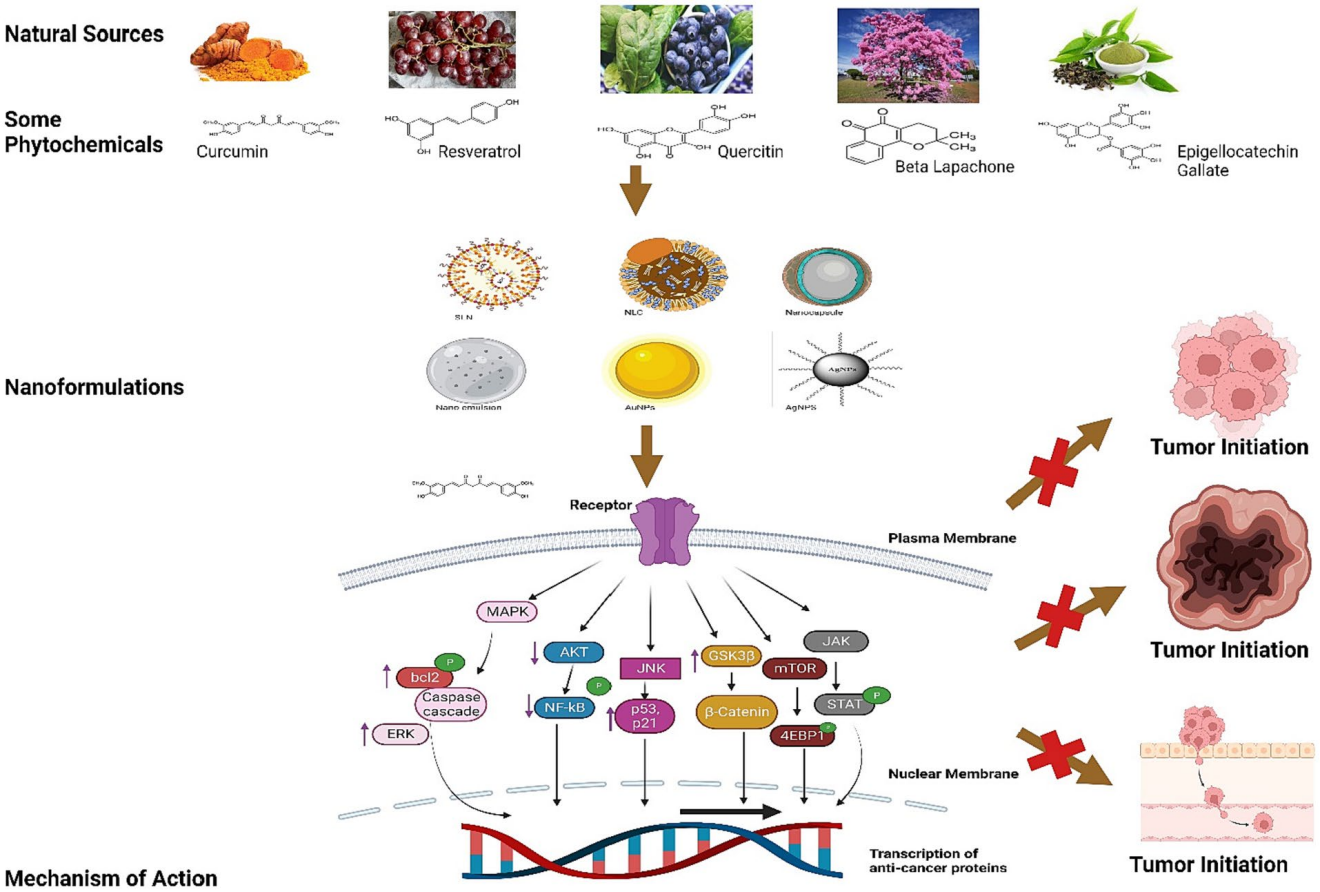
This illustration explains the mechanism of Resveratrol, Curcumin, Quercetin, Beta Lapachone, Epigallocatechin Gallate and others chemical structures in inhibition of tumor imitation.

**Figure 4:**
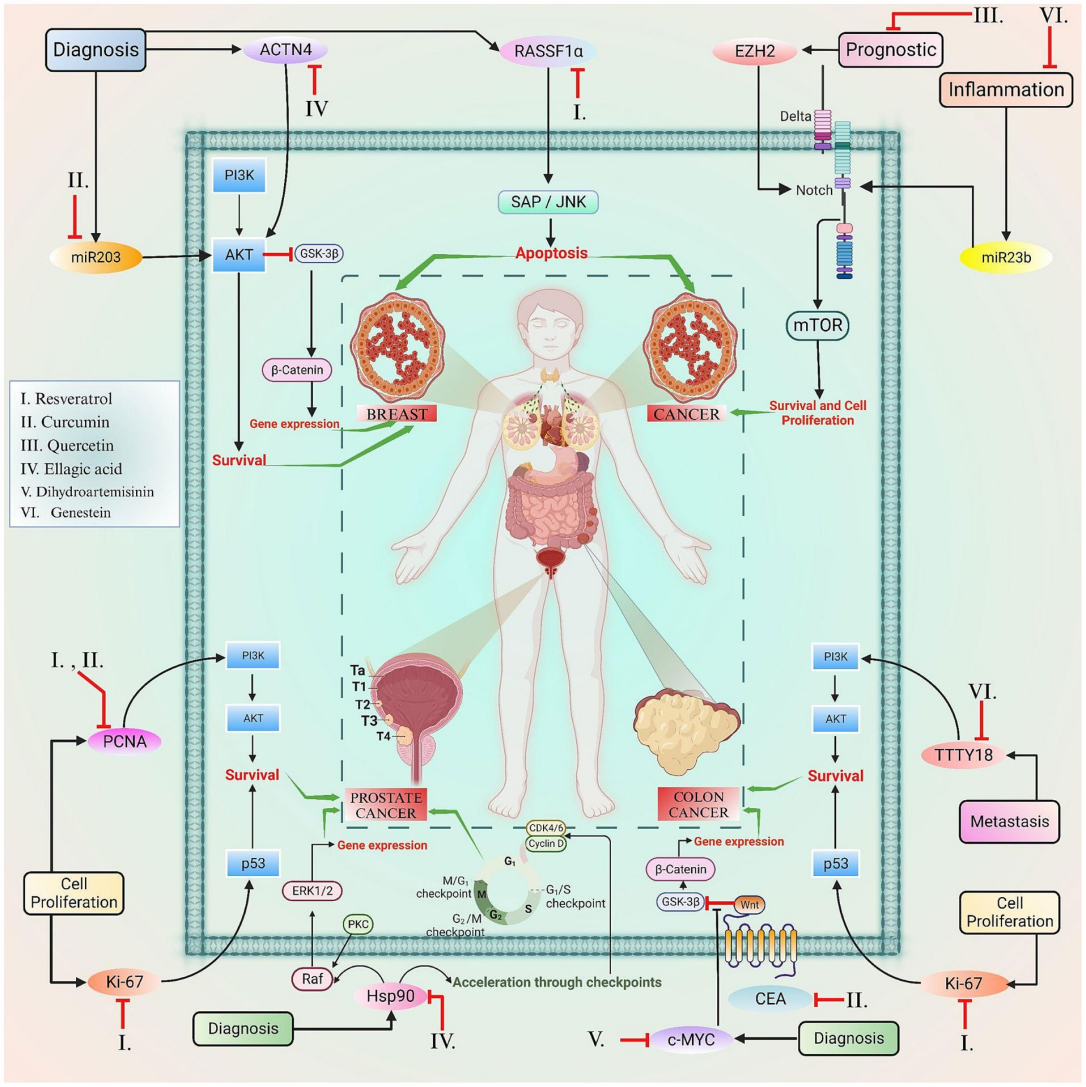
This figure elucidated the correlation between the certain biomarkers and phytoconstituents involved in various types of cancer.

The mechanism of action by these nutraceuticals and their formulations has been explained in Figures 3, 4.

## 5 Types of cancer biomarkers and receptors present and the targeting strategies

Cancer continues to be among the most devastating diseases and contributes significantly to global mortality. Cancer is usually classified as a benign or malignant tumor. Malignant tumors are particularly vulnerable and manage to propagate throughout the body through the lymphatic or circulatory systems, while benign tumors are discrete and demonstrate restricted development with fewer dangerous characteristics and indications (119). These cancerous tumors invade healthy tissue and fuel their continued growth and dissemination of necrotic cells by forming new blood vessels. Therefore, unique chemicals are produced by benign and malignant cells known as cancer markers or tumor markers. However, the level of tumor marker in cells with cancer exceeds that in healthy cells. Tumor markers are therefore significant prognostic or diagnostic tools for various forms of cancer, since some are associated with a single type of cancer while others are associated with two or more types of cancer (120, 121). The precise and dependable identification of these tumor markers is critical in early detection, which greatly enhances the chances of successful therapy and recovery. Most cancer markers may be described as proteins (122).

### 5.1 CEA (carcinoembryonic antigen)

Gold and Freedman initially identified CEA in tissue samples of human colon cancer (123). The CEA molecule is a glycosyl phosphatidylinositol (GPI)-cell surface anchored glycoprotein, a complex macromolecule comprising about 50% carbohydrate and around 200 kDa in weight that is created throughout fetal development but culminates before birth (124–127). CEA is a promising serum tumor marker for colorectal and other malignancies due to its stability, limited expression in normal adult tissue, and high expression in malignant tumors (128). CEA binds to its receptor (CEAR), a member of the hnRNP M family of RNA-binding proteins, to exercise its actions in the liver (129, 130). CEA attaches to hnRNP M on Kupffer cells in the liver, activating those cells and leading to the generation of cytokines that cause inflammation, such IL-1, IL-10, IL-6, and TNF-α (131, 132) The role of these cytokines is to provide protection to cancer cells from the harmful effects of NO and ROS. Additionally, they have an impact on amplification of adhesion molecules, which are found on the hepatic sinusoidal endothelium (133). Therefore, HnRNP M signaling regulates the stimulation of beta-adrenergic receptors in Kupffer cells. Cells produce less TNF-α and more IL-10 and IL-6 in response to the ***β***-adrenergic receptor agonist terbutaline, leading to CEA activation (134).

### 5.2 HER2

The initiation of targeted therapy for Human Epidermal Growth Factor Receptor-2 (HER-2) may be traced back to the year 1962 when Stanley Cohen made a significant breakthrough by identifying the protein known as Epidermal Growth Factor (EGF), which was responsible for the expansion of the mouse incisors and eyelids (135, 136). The first tyrosine kinase, epidermal growth factor receptor (EGFR), was identified in 1978, sparking the discovery of the HER2 gene in 1984 (137, 138). HER2 belongs to a family of four cell surface receptors that influence the development and division of cells via pathways of signal transduction (139). Extracellular ligand-binding domains, transmembrane domains, and intracellular tyrosine kinase domains have been recognized in HER receptors. When ligands attach to the HER proteins, the receptors may homodimer or heterodimerize, triggering downstream signaling pathways that encourage cell proliferation and development while eliminating apoptosis (140). Although HER2 does not have a specific ligand, it serves as a preferred dimerization partner for HER3 and the other HER proteins (141). Overexpression of HER2 induces human breast malignant growths; however, HER2 signaling and transforming activities are engaged in the regulation of normal breast growth and development, which is linked with an undesirable prognosis (142, 143). The overexpressed HER2 receptor is a tempting target for several anticancer drugs. Patients with HER2+ cancer have seen an increase in survival rates with the use of targeted anti-cancer drugs such as trastuzumab, pertuzumab, and,presently, tucatinib and trastuzumab deruxtecan (144).

### 5.3 CA 125 (cancer antigen 125)

An antigenic marker CA 125 also recognized as Mucin 16, was initially proposed as a tumor marker for the diagnosis of epithelial ovarian cancers in 1980’s (145). It was discovered with the utilization of the monoclonal antibody OC 125, which was created by immunizing mice with epithelial ovarian cancer cell lines (146). It is a glycoprotein cell-surface antigen identified in tissues that originate from coelomic epithelium, which include the ovary, fallopian tube, peritoneum, pleura, pericardium, colon, kidney, and stomach (147). In order to identify CA 125, malignant ovarian or endometrial tissue samples may be employed in tissue-based examinations or by serological testing (148). It is not advised to use CA125 in a wide screening since it fails to analyze over half of early malignancies and may add unnecessary stress to patients’ therapy processes and lead to inappropriate medical decisions (149). However, there have been several studies conducted whereby a variety of methods have been shown to improve the diagnosis reliability of CA125. Combining CA125 with other biomarkers and improving CA125 detection is a possible approach for early- stage diagnosis of ovarian cancer, particularly when Glyco-Variant CA125 testing is employed (150–152). Survivin, calcitonin, vimentin, β-2 microglobulin, Myc and A1B1 and Caspase-3, targeting bladder, thyroid, kidney, Multiple myeloma and lymphoma, Hepatocellular carcinoma, Gastric carcinoma are some additional markers used for determination of screening, diagnosis, prediction as well as recurrence (153–157). FDA has granted authorization for the use of urine biomarkers, namely BTA and nuclear matrix protein-22, in clinical settings as diagnostic indicators for bladder cancer (158, 159). No single biomarker is expected to have sufficient confidence to govern treatment choice in determining illness outcome. Therefore, small panels of 6–10 markers may be adequate for precise molecular staging, allowing the prediction of metastatic development and the prompt implementation of systemic treatment in cancer prognosis.

Certain nutraceuticals and their phytoconstituents have been found to have a promising role in the regulation of biomarkers associated with various hallmarks of cancer. For instance, the involvement of miRNA in gene mutation, the assessment of Ki-67 and PCNA in cellular proliferation, the significance of p53 in diagnosis and its role in regulating tumor suppressing genes, and additional relevant factors are extensively elaborated upon in Table 2.

**Table 2:** Regulatory effects of phytoconstituents on various biomarkers associated with various cancer types.

S.No.	Phytoconstituent	Type of cancer	Biomarkers	Application of biomarker	Outcome	Reference
1.	Resveratrol	Colorectal	Ki-67	Cancer proliferation	It leads to downregulation of Ki-67 levels on the growth and progression of malignancy.	Patel et al. (160)
			Caspase-3	Apoptosis	It led to an increase in cleaved caspase-3 levels within malignant hepatic tissue.	Howells et al. (161)
		Breast	RASSF1α	Detection and prognosis	The reduction of methylation levels in the RASSF-1α gene on administration of resveratrol.	Zhu et al. (162)
		Prostate	Ki-67	Cancer proliferation	Significant reduction in the levels of cellular proliferation markers, specifically Ki67.	Singh et al. (163)
			PCNA	Cancer proliferation	Reduction in the levels of PCNA, a well-established marker of cell survival, has been observed upon exposure to resveratrol.	Singh et al. (163)
2.	Curcumin	Breast	miRNA-203	Diagnosis	It leads to an increase in miR-203 which shows promising therapy against cancer by regulating epigenetic code, a key mechanism involved in the development and progression of breast cancer.	Khan et al. (164)
		Bladder	Ki-67	Cancer proliferation	The findings of this study demonstrate a notable reduction in Ki-67 expression.	Kamat et al. (165)
		Prostate	PCNA	Cancer proliferation	Significant reduction in PCNA levels, which subsequently leads to the activation of apoptosis of malignant cells.	Barve et al. (166)
		Pancreatic	miRNA-22	Diagnostic	The up-regulation of miRNA-22, induced by curcumin, has been found to exhibit inhibitory effects on the growth of pancreatic cancer cells.	Khan et al. (164)
		Hepatic	miRNA-21	Diagnosis	The decline in miR-21 has been found to play a crucial role in facilitating the regulation of the cell cycle and apoptosis by elevating PTEN and PDCD4 Protein level in Hepatic cancer.	Khan et al. (164)
3.	Quercetin	Breast	EZH2	Prognostic	The inhibition of EZH2 has been found to have a substantial impact by effectively blocking the Notch-1 and P13K/Akt signal pathways.	Cao et al. (167)
		Liver	AFP	Diagnosis	The intervention demonstrated significant efficacy in reducing the incidence of AFP rates.	Abdu et al. (168)
		Prostate	Hsp90	Diagnosis	The stimulation of cell death and caspases through the inhibition of Hsp90 was observed.	Aalinkeel et al. (169)
		Thyroid	Pro-NAG1	Diagnosis	The induction of apoptosis was observed to be mediated through the selective upregulation of pro-NAG-1 expression.	Hong et al. (170)
4.	Beta lapachone	Breast	NQO1	Prognosis	The inhibitory effects of β-lapachone on the Akt/mTOR signaling pathway were observed through the downregulation of NQO1.	Yang et al. (171)
		Pancreatic	NQO1	Prognosis	The administration of β-lapachone resulted in a significant reduction in NQO-1 levels.	Silvers et al. (172)
5.	Epigallocatechin	Breast	Akt	Indication of regulation of P13K/mTOR pathway	The inhibitory effects of polyphenol treatment on AKT were observed at both the RNA and protein levels resulting in cancer’s attenuation.	Thangapazham et al. (173)
			p53	Diagnosis	It inhibits the development of malignant breast cells and induces apoptosis through deregulating the P53/Bcl-2 pathway.	Huang et al (174)
		Hepatic	Ki-67 and PCNA	Cell proliferation	A significant decrease in the expression levels of PCNA and Ki-67 which results in inhibitory effects on cellular proliferation in the liver.	Sojoodi et al. (175)
		Bladder	PCNA	Cell proliferation	A significant decrease in the biomarker levels, which was found to be dependent on the dosage administered.	Piwowarczyk et al. (176)
			IL-1β	Inflammation	IL-1β suppression decreases formation of ROS, AP-1/NF-kB, and cancer cell migration.	Sah et al. (177)
		Colon	ACF	Prognosis	The formation of ACF was found to be markedly suppressed, as evidenced by a notable reduction in their size within cancer cells.	Zhong et al. (178)
6.	Ellagic acid	Breast	Actinin-4 (ACTN4)	Early diagnosis and prediction	The inhibitory effects on ACTN4 led to the suppression of cancerous cell multiplication and colony formation by declining β-catenin proteasome.	Wang et al. (84, 85)
		Hepatic	AFP	Diagnosis	There was a substantial reduction in the concentration of AFP.	Zaazaa et al. (179)
		Oral	Birc5	Diagnosis and prognosis	It effectively impedes tumor cell proliferation and hinder disease progression by diminishing anti-apoptotic associated Birc5 marker.	Oghumu et al. (180)
7.	Daidzein	Breast	Caspase-9	Apoptosis	Increase in caspase-9 activity by 32.2% upon exposure to Daidzein, leading to the induction of apoptosis.	Choi et al. (181)
8.	Dihydroartemisinin	Brain	HSPA-5	ER stress	HSPA5, a key marker of ER stress was increased which inhibits the PERK/ATF4 pathway.	Chen et al. (182)
		Colon	c-MYC	Diagnosis	A decrease in the levels of the c-MYC protein, a pivotal modulator of several cellular processes, through the proteasome pathway.	Lu et al. (183)
		Hepatic	ANGPTL 2	Cellular Senescence	The inhibition of multiplication and invasion in glioma cells has been attributed to the decline in ANGPTL2 levels, which effectively suppresses the ERK/MAPK pathway.	Wu et al. (184)
		Ovarian	PDGFRα	Prognostic	The interruption of PDGFRα by DHA results in interruption of cell growth, multiplication, and apoptosis in malignant cells.	Li et al. (185)
9.	Triptolide	Adeno (Stomach)	CXCR4	Prognostic	The therapeutic targeting of CXCR4 by triptolide holds significant promise in the realm of cancer treatment.	Qiu et al. (186)
		Pancreatic	KRAS	Diagnosis	A significant reduction in KRAS mutation levels in cancer cells, leading to a notable antitumor effect.	Kim et al. (187)
10.	Genistein	Breast	miR-23b	Inflammation	It upregulates miR-23b effectively inhibits cell migration and metastasis.	Avci et al. (188)
		Colon	TTTY18	Cancer development/metastasis	A significant reduction in tumor volume, accompanied by decrease in both TGF-β1 contents and TTTY18.	Chen et al. (189)
		Ovarian	Malondialdehyde	Oxidative stress	The intervention resulted in a decrease in the marker and level of NFkB, while simultaneously increasing Nrf2 and Bax in cancer cells.	Sahin et al. (190)

## 6 Nanoformulation for anticancer therapy

Phytoconstituents, the bioactive components of plants, have demonstrated promise in cancer treatment due to their natural potential to combat this disease. In order to address the constraints inherent with the intrinsic suboptimal physicochemical characteristics of these compounds, researchers have turned to nanotechnology to enhance their delivery and therapeutic effect (191). Nanoformulation is a promising approach for improving the delivery and efficacy of phytoconstituents in cancer treatment. Encapsulation is a significant process that warrants meticulous examination. To encapsulate a compound effectively, it is crucial to take into account the molecular structure, which correlates with its size and solubility; the properties of the carrier, such as the materials comprising it; the specific application, particularly administration routes in clinical settings; and the matrix material for varied industrial applications (192). Nano formulation offers several advantages for the targeted therapy of nutraceuticals, including:

1. Enhanced bioavailability: Nano-sized particles have a larger surface area, allowing for better solubility and absorption of nutrients in the body, resulting in improved bioavailability.
2. Targeted delivery: Nano-formulation enables precise delivery of nutraceuticals to specific cells or tissues, ensuring optimal therapeutic effect and reduced side effects.
3. Controlled release: By encapsulating the nutraceuticals in nano-carriers, it is possible to achieve sustained and controlled release, thus maintaining consistent levels of nutrients in the body over an extended period.
4. Improved stability: Nano-formulation can protect sensitive nutraceutical compounds from environmental factors such as oxidation, light, and temperature, prolonging their shelf life and retaining their therapeutic properties.
5. Increased solubility: Many nutraceuticals are poorly soluble in water, making them difficult to absorb by the body. Nano-formulation could help improve their solubility and aid in better absorption.
6. Reduced toxicity: The targeted nature of nano-formulation can minimize harmful interactions with other molecules or healthy cells, thereby reducing potential toxicity concerns associated with higher doses of certain nutraceuticals.

Overall, nano-formulation presents a promising strategy for enhancing the efficacy and safety of nutraceutical therapies by ensuring targeted delivery, improved bioavailability, controlled release, and increased stability (193, 194).

### 6.1 Solid-lipid nanoparticles

**Figure 5:**
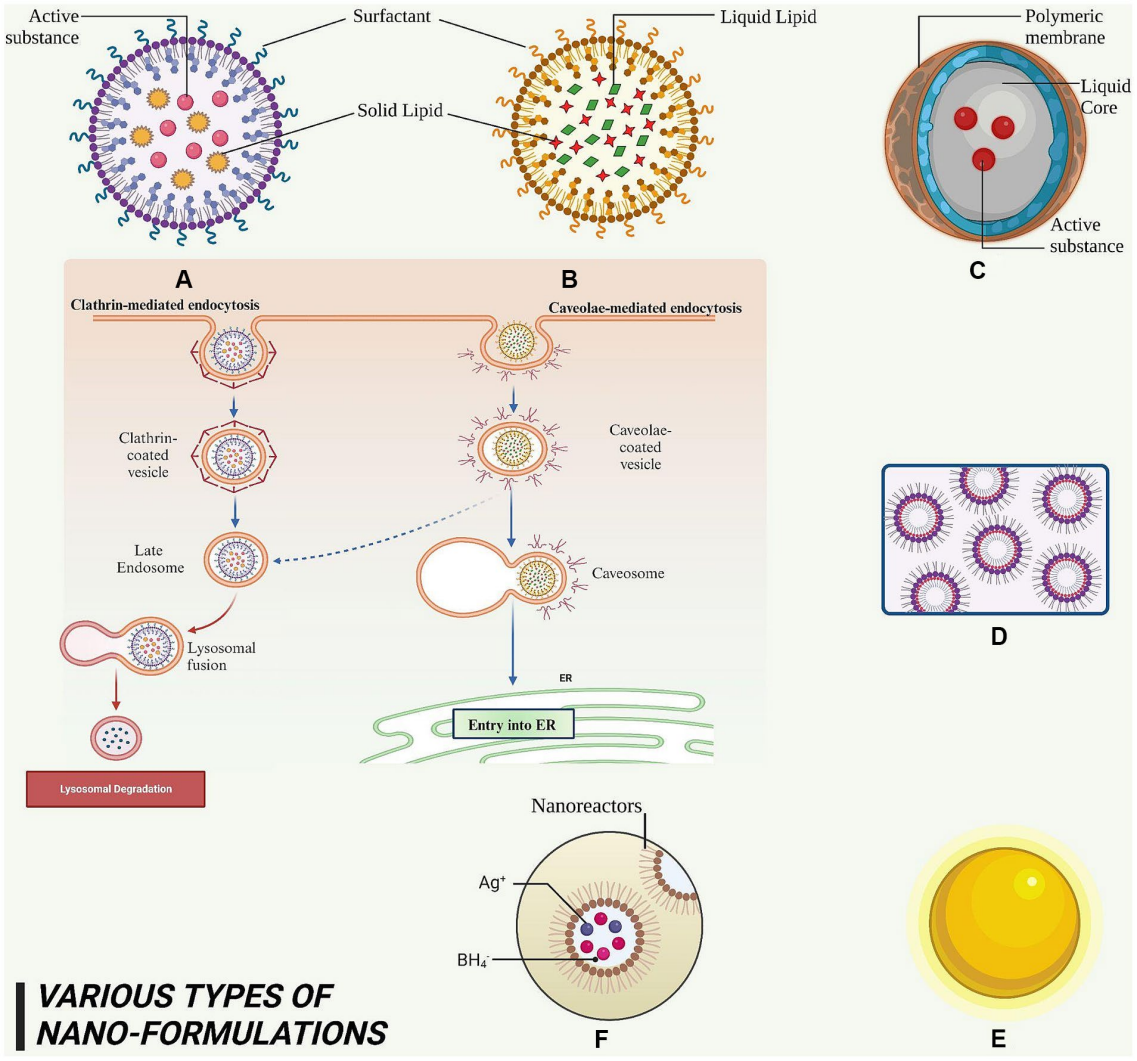
This figure elucidated diversity of nanoformulations and their constituent elements that serve for the administration of phytoconstituents in anti-cancer therapy. **(A)** SLN – it is a nanoscale particle with dimensions starting from 50 nm, composing of solid lipids. **(B)** NLC – this refers to a type of nanoparticle with a size range of approximately 10–1,000 nm, composed of both solid and liquid lipids. **(C)** Nanocapsules – they are nano-sized which exhibits the range of 100–1,000 nm and consist of a polymeric membrane. **(D)** Nano emulsions – it is a colloidal dispersion with droplet sizes typically ranging from 100 to 600 nanometers. **(E)** AuNPs – it exhibits a size range of approximately 5 to 400 nm. **(F)** AgNPs – it exhibits a size range of approximately 1–100 nm.

Solid-lipid nanoparticles (SLNs) are diminutive particles with sizes ranging between 50 and 100 nm as shown in Figure 5A. Liquid lipids, or oils, have traditionally been utilized solely for nano- emulsions. Typical lipids used in SLN preparation encompass glycerides, sterols, partial glycerides, fatty acids, and waxes, rendering a highly lipophilic lipid matrix. Numerous administration methods for SLNs include oral, parenteral, rectal, nasal, ocular, and topical routes. SLNs possess straightforward and cost-effective manufacturing processes suitable for large-scale industries (195, 196). Furthermore, they serve as groundbreaking occlusive topicals exhibiting controlled release without adverse toxic effects upon topical application. SLNs offer an innovative drug delivery mechanism that evades immune system clearance while accommodating enhanced bioavailability of poorly water-soluble pharmaceuticals and facilitating reduced dose frequency for improved patient compliance (197). Additionally, they improve drug solubility, residence time, and lymphatic absorption and have been employed to deliver diverse phytoconstituents with anti-cancer potential having low bioavailability and varying lipophilicity and hydrophilicity, as demonstrated in Table 3.

**Table 3:** Anti-cancerous application of natural occurring therapeutic molecules based nanoformulations.

Types of nano approach	Components of nano formulation	Phytoconstituents / combinations of phytochemicals	Method of synthesis	Application and cell line/model	Outcome	Reference
SLN	Hydroxypropyl β-cyclodextrin and gelucire	Curcumin and Resveratrol	Sonicatio n and freeze drying	*in-vitro* study on HCT-116 cell lines	The nanoformulati on exhibits a significant increase in halting cell multiplication of colon tumor cells compared to the insoluble mixture	Gumireddy et al. (198)
NLC	Fluvastatin	Egallic acid and Alpha lipoic acid	Hot emulsification–ultrasonication method	*in-vitro* study on PC-3 prostate cancerous cell line	It results in cell death more effectively compared to the combination without NLC, through the mechanisms of cell cycle interruption and minimized caspase 3 expression	Fahmy (199)
NLC	Fluvastatin	Egallic acid and Alpha lipoic acid	Hot emulsification– ultrasonication method	*in-vitro* study on PC-3 prostate cancerous cell line	It results in cell death more effectively compared to the combination without NLC, through the mechanisms of cell cycle interruption and minimized caspase 3 expression	Fahmy (199)
NLC	Glycerol monostearate, soybean phosphatidylcholi ne and PEG	Curcumin and Etoposide	Solvent injection technique	*in-vitro* study on SGC7901 and *in-vivo* experiment in BALB/c nude mice	The animal model demonstrated better anti cancer efficacy against gastric carcinoma at a lower dose when administered combination drug-loaded NLCs compared to single drug- loaded NLCs	Jiang et al. (200)
NLC	Tristearin and TPGS	Genistein	Solvent emulsifica tion and evaporatio n method	*in-vitro* study on PA-1	The anti-tumor activity was demonstrated on PA-1 cell lines is more desirable to that of unmodified Genistein	Mittal et al. (201)
NLC	Glycerol monostearate and soya lecithin	Genistein and Curcumin	Nanoemul sion method	*in-vitro* study on PC-3 cell line	The findings of this investigation illustrate the improvement in the inhibition of cell development resulting in the encapsulation of curcumin and genistein within NLCs	Aditya et al. (202)
Nanocap sules	Poly (ɛ- caprolactone), capric/caprylic triglyceride and sorbitan monostearate	Resveratrol	Interfacial deposition	*In-vitro* study on C6 rat glioma cell line *In-vivo* study on implanted C-6 cells in rat	The experimental formulation demonstrated a more pronounced reduction in the survival of C6 glioma *in vitro* as compared to the impact of resveratrol in solution. It declined via cell death and the arrest of cell cycle progression at an early stage in the S and G1 phases. The experiment demonstrated selectivity for cancer cells and yielded a noticeable decrease in tumor dimensions	Figueiró et al. (203)
Nano Emulsion	Capmul MCM and tween 80	Resveratrol and Tamoxifen	Homogeni zation	*In-vitro* study on MCF-7	Its therapeutic efficacy is significantly higher than that of the traditional preparation. It also proved superior internalization on the MCF-7 cell line	Shrivastava et al. (204)
AuNPs	0.1 M sodium hydroxide solution, SDS and ethyl alcohol absolute.	Genistein	One pot synthesis	*In-vitro* studies on A549 lung cancer cell line and HTB-140 cell line	This formulation exhibited superior cytotoxicity against cancer cells in comparison with untreated genistein	Stolarczyk et al. (205)
AuNPs	Trypsin–EDTA and TRIzol	Epigallocatechin gallate	Reduction methods	*In-vitro* studies on various cell lines including MCF-10A, HPNE, PC3 and A375SM cell line.	It has preferential affinity towards malignant cells and a rapid stimulation of cell death, coupled with a strengthened efficacy in the inhibition of NF-κB	Chavva et al. (206)
AgNPs	Chitosan, soluble starch and tween 80	Ellagic acid and Alginate Silver	Magnetic stirring and centrifuga tion technique s	*In-vitro* studies on normal mouse fibroblast cell and MCF-7 breast cancer cell line	It illustrates cytotoxicity against MCF- 7 cells based on its increase in concentration and displays a high degree of sensitivity towards them	Hussein-Al-Ali et al. (207)

### 6.2 Nanostructured lipid carriers

Nanostructured lipid carriers (NLCs) are solid and liquid lipid mixtures combined with an aqueous phase containing surfactants in varying combinations to produce an unstructured solid- lipid matrix as shown in Figure 5B. Lipids are the predominant constituent of NLCs and exert influence on crucial factors such as loading limit, duration of action, and composition durability. Lipids that are able to decompose, are not toxic, and physiologically acceptable, are widely acknowledged as safe (GRAS), have been suggested as suitable candidates for the development of lipid nanoparticles. During NLC synthesis, incorporation of liquid lipids is essential for creating an amorphous lattice within the solid core matrix. Solid lipids may encompass triglycerides (tristearin), fatty acids (stearic acid), waxes (carnauba wax), and steroids (cholesterol). Liquid lipids comprise medium-chain triglycerides (such as miglyol 812), natural oils (such as olive oil), fatty acids (such as oleic acids), and other oily substances (such as paraffin oil) (208, 209). NLCs represent cost-effective approach that enhance solubility and bioavailability of drugs and phytoconstituents, delivering them to target sites with optimal efficiency, contributing to improved therapeutic efficacy, and preventing therapeutic molecule expulsion during storage. NLCs facilitate prolonged release, making them ideal for medications with brief half-lives. Furthermore, they display increased permeability across cellular membranes, leading to more effective drug delivery. They can also be customized to meet the requirements of various medicines, making them suitable for multiple applications. The distinct composition of NLCs enables researchers to explore their potential in transforming medical delivery through numerous administration routes such as topical, nasal, inhalation, and oral methods (210, 211). It has been extensively utilized in cancer research as demonstrated in Table 3.

### 6.3 Nanocapsules

Nanocapsules are minuscule particles sized between 100 and 1,000 nanometers as shown in Figure 5C. Each nanocapsules contains a core that carries one or more active chemicals. This core is enveloped by an oil-based surfactant selected for its high drug solubility and low membrane compatibility. Surrounding the core structure is a nontoxic polymer membrane composed of biodegradable polyester that facilitates proper drug delivery rate and timing without adverse effects (212, 213). Commonly used biodegradable polymers for forming the protective core layer include poly lactic acid, poly(D, L-glycolide), poly(D, L-lactide-co-glycolide), and poly(cyanoacrylate). The primary objective is to boost drug administration rate and duration while ensuring patient safety (214). Owing to their larger surface area, they can effectively transport drugs through biological membranes, enhancing bioavailability and offering controlled drug release. Recent studies examining the outcomes of nanoencapsulation show promising results in addressing key drug-related issues such as solubility and permeability (215). They enable a sustained and targeted delivery of phytoconstituents to cancer cells and reduce side effects on healthy tissue as shown in Table 3.

### 6.4 Nanoemulsions

Nanoemulsions, also referred to as “mini emulsions” or “ultra-fine emulsions,” are o/w or w/o dispersions comprising spherical droplets ranging from 100 to 600 nm in size as shown in Figure 5D. The oil phase consists of triacylglycerols, diacylglycerols, monoacylglycerols, and free fatty acids, with long-chain triacylglycerols being preferred due to their abundance in nature and affordability. Meanwhile, the aqueous phase contains a polar solvent (water) and cosolvent (carbohydrates, proteins, alcohol, etc.) (216). Nanoemulsions offer enhanced properties compared to conventional emulsions and microemulsions. To avoid instability caused by coalescence and flocculation, emulsifying agents can be added. The small size of nanoemulsions endows them with several advantages over traditional emulsions and microemulsions. For example, they can be administered orally as the stabilizing agents are non- irritating and nontoxic. Furthermore, they can be incorporated into various dosage forms such as syrups, sprays, foams, and creams. The therapeutic potential of molecules encapsulated within nanoemulsions is enhanced due to increased solubility, permeability, and dissolution profile. These nanoemulsions exhibit antimicrobial properties, allowing for a reduction in substances that may cause undesired toxicity (217, 218). Moreover, they enhance penetration of the active compound through biological membranes and have proven to be an effective therapy to treat cancer as evident in Table 3.

### 6.5 Gold nanoparticles

Nanoparticles of noble metals, possessing minute dimensions, play an essential role in diagnosing and treating various health conditions. Ranging between 1 to 100 nm in size as shown in Figure 5E, these metal nanoparticles demonstrate superior catalytic performance and biocompatibility when compared to free-form pharmaceuticals. Their effectiveness depends on their shape. Gold nanoparticles (AuNPs) are generally acknowledged as being very influential in the field of targeted supply of medications at specific sites owing to their exceptional chemical stability, non-cytotoxic nature, and extensive surface area (219). Existing in diverse shapes (e.g., nanorods, nanotriangles, nano prisms, and hexagonal platelets), these particles typically measure less than 100 nm. Their properties facilitate strong binding with biomarkers for targeted drug delivery. They can penetrate specific cell walls within the body, exhibiting their antibacterial capabilities effectively. The exceptional catalytic activity of AuNPs may be attributed to their excellent surface area-to-volume proportion and interface-dominated characteristics – contributing to the development of nanosized electrocatalysts and optimizing catalytic activity/selectivity (220, 221). Additionally, AuNPs show intrinsic cytotoxicity against cancer cells through mechanisms such as oxidative stress induction or mitochondria dysfunction as illustrated in Table 3.

### 6.6 Silver nanoparticles

Silver nanoparticles (AgNPs) are prevalently utilized globally, with approximately 313 silver nanoparticle-based products available commercially. They possess distinct advantages over other noble metals such as high thermal conductivity, chemical stability, nonlinear optical behavior, large surface area, and high catalytic activity. Notably, AgNPs can be applied in both solid and liquid forms. Ranging between 2 nm and several hundred nanometers in size, silver nanoparticles display color variations depending on their dimensions (222) as shown in Figure 5F. The primary motivation for using these particles is taking advantage of their antimicrobial properties; they are effective against 16 major bacterial species. Optimal antimicrobial activity occurs when silver nanoparticles are combined with nitrates (223). They can be synthesized in various forms, including nanowires, tabular prisms, cubes, octahedra, and pyramids. The shape and size of AgNPs are influenced by factors during synthesis such as temperature, pH solution, silver concentration in synthetic production, and substances employed in biological synthesis (224). Like gold nanoparticles, silver nanoparticles can also serve as carriers for anticancer phytoconstituents. They exhibit inherent cytotoxicity to cancer cells by building up ROS, leading to apoptosis or necrosis. Silver nanoparticles can be used in combination therapy with phytoconstituents for synergistic anticancer effects as justified in Table 3.

## 7 Applications of nano nutraceuticals in anticancer therapy

The rapidly evolving field of nanotechnology has opened new doors for the fields of cancer therapy and nutraceuticals. The integration of nanotechnology in nutraceuticals for cancer treatment has a significant potential for improving patient outcomes. Applications of nano nutraceuticals in anticancer therapy include targeted drug delivery, enhanced bioavailability, increased solubility, improved stability, and mitigation of unfavorable side effects (225–227). Phytoconstituents such as curcumin, quercetin, and resveratrol are becoming more effective in cancer treatment due to nanotechnology advancements that allow for better absorption and therapeutic efficacy. Various studies have been discussed below demonstrating the specific examples of phytoconstituents in nano nutraceuticals showing promising results along with improvements and applications of this technology in anti-cancer therapy.

## 8 The promising future of anticancer treatment with nutra-nanotechnology

Nano-formulation approaches bolsters the positive prospects of nutraceuticals in both avoidance and cure of cancer. Nevertheless, the existing limitations in dissolution rate of phytoconstituents have resulted in a restricted oral bioavailability, thereby hindering the exploration of its complete range of capabilities. Nano-nutraceuticals not only enhance the bioavailability of these compounds by improving their solubility and permeability but also contribute to increased stability, thereby ensures to pave the way for future advancements in this field. As they are derived from natural sources, they are considered safer and possess fewer side effects compared to conventional chemotherapeutic drugs. Therefore, nano-engineered nutraceuticals could potentially revolutionize the landscape of cancer prevention and therapy by offering a safe, effective, and targeted approach to combating this deadly disease. To harness its full potential, it is essential to conduct comprehensive clinical trials to validate their safety and efficacy across different cancer types. This will also help determine optimal product formulations for various cancers while ensuring that minimum effective doses deliver maximum therapeutic benefits with minimal adverse effects. Furthermore, trial will also investigate the untapped synergistic potential of this innovative approach in conjunction with approved therapies for the management of cancer (228).

## 9 Conclusion

Since time immemorial, herbs have served as natural remedies for an array of physiological ailments. Their invaluable contributions to human health and disease treatment have been acknowledged in traditional medical texts. Recent research has delved into incorporating herbs and their functional components into foods, examining their interactions with dietary constituents. Herbal medicine is believed to be the primary healthcare choice for approximately 80% of the global population. In the realm of cancer therapy, nutraceuticals – which possess anti- inflammatory, antioxidant, antimicrobial, anti-carcinogenic, and hepatoprotective properties – have garnered increased interest. Their significant potential lies in activating antioxidant pathways and inhibiting inflammatory signaling pathways, offering a non-invasive method to treat or prevent certain chronic illnesses. Through the enhanced delivery of bioactive compounds, they improve therapeutic efficacy and reduce potential toxicity associated with traditional anticancer treatments. As research on nano-based nutraceuticals continues to progress, it is evident that the development of novel strategies for targeted delivery, controlled release systems, and improved biocompatibility will further revolutionize cancer treatment. While more extensive preclinical studies are essential to better understand their toxicity and long-term effects fully, nano-based nutraceuticals hold a significant position in the ongoing advancements in the field of oncology. Recent developments have also explored the therapeutic efficacy and cost-effectiveness of nanotechnology-based nutraceuticals as a viable alternative to traditional preservatives. Current innovations are focused on formulating economically feasible nano-nutraceutical solutions to further bolster their applicability in cancer therapy. Scientists anticipate that these innovative approaches will not only complement existing therapies but also pave the way for future breakthroughs in cancer management and prevention.

## Author contributions

MS: Writing – original draft. Smriti: Writing – original draft, Writing – review & editing. SG: Formal analysis, Supervision, Writing – review & editing. PB: Writing – review & editing. SS: Data curation, Methodology, Supervision, Writing – review & editing. SP: Formal analysis, Funding acquisition, Resources, Visualization, Writing – review & editing. SR: Writing – original draft, Writing – review & editing. JB: Writing – original draft, Writing – review & editing. PM: Conceptualization, Formal analysis, Writing – original draft, Writing – review & editing. SM: Funding acquisition, Resources, Visualization, Writing – review & editing. PS: Funding acquisition, Resources, Visualization, Writing – review & editing.
